# Design Rationale and Performance Evaluation of the Wavelet Health Wristband: Benchtop Validation of a Wrist-Worn Physiological Signal Recorder

**DOI:** 10.2196/11040

**Published:** 2018-10-16

**Authors:** Onur Dur, Colleen Rhoades, Man Suen Ng, Ragwa Elsayed, Reinier van Mourik, Maulik D Majmudar

**Affiliations:** 1 Wavelet Health Mountain View, CA United States; 2 Biomedical Engineering San Jose State University San Jose, CA United States; 3 Healthcare Transformation Lab Massachusetts General Hospital Harvard Medical School Boston, MA United States

**Keywords:** wearable electronic devices, vital signs, monitoring, ambulatory, photoplethysmography, benchmarking

## Abstract

**Background:**

Wearable and connected health devices along with the recent advances in mobile and cloud computing provide a continuous, convenient-to-patient, and scalable way to collect personal health data remotely. The Wavelet Health platform and the Wavelet wristband have been developed to capture multiple physiological signals and to derive biometrics from these signals, including resting heart rate (HR), heart rate variability (HRV), and respiration rate (RR).

**Objective:**

This study aimed to evaluate the accuracy of the biometric estimates and signal quality of the wristband.

**Methods:**

Measurements collected from 35 subjects using the Wavelet wristband were compared with simultaneously recorded electrocardiogram and spirometry measurements.

**Results:**

The HR, HRV SD of normal-to-normal intervals, HRV root mean square of successive differences, and RR estimates matched within 0.7 beats per minute (SD 0.9), 7 milliseconds (SD 10), 11 milliseconds (SD 12), and 1 breaths per minute (SD 1) mean absolute deviation of the reference measurements, respectively. The quality of the raw plethysmography signal collected by the wristband, as determined by the harmonic-to-noise ratio, was comparable with that obtained from measurements from a finger-clip plethysmography device.

**Conclusions:**

The accuracy of the biometric estimates and high signal quality indicate that the wristband photoplethysmography device is suitable for performing pulse wave analysis and measuring vital signs.

## Introduction

### Wearable and Connected Devices in Digital Medicine

Wearable and connected health devices have been increasingly popular with the general population to track metrics related to their personal health and wellness such as vital signs, activity, and sleep [1]; a purchase of over 300 million wearable devices was reported in 2017 worldwide [2]. Not surprisingly, wearables have recently been adopted by clinical researchers to paint the most complete picture of patients’ health and well-being by offering continuous, long-term, and multiparametric monitoring [3]. Leveraging mobile, wearable and connected devices, and cloud computing and machine learning algorithms, the modern era of health care—known as digital health—promises new and better ways to screen, diagnose, manage, and treat patients, thereby improving health-related outcomes and decreasing the cost of health care delivery. It is well known that measuring health is the key to (1) improve screening and preventative methods; (2) optimize disease management, drug titration, and adherence to therapy; and (3) prevent hospitalization and reduce adverse events. Wearable and connected devices offer a promising solution to streamline health data collection needs and address the lacking personalized oversight in the current health system.

Clinical research organizations and pharmaceutical companies have already started embracing digital health. Recently, several large-scale research initiatives involving parties across government agencies, health institutions, and private technology and pharmaceutical companies [4-6] have announced collecting biological, psychological, and social signals and biometrics derived from these signals in longitudinal studies to understand underlying risk factors leading to disease and hospitalizations. There is also a growing interest in the pharma community to use mobile, wearable, and connected health technologies to improve the operational efficiency during the product development cycle and to accelerate bench-to-bedside translational science. Digital operational efficiency here refers to the use of digital health to increase patient adherence, trial data collection speed, and efficiency, thereby reducing the time and cost to market. Electronic patient-reported outcomes and remote patient monitoring are good examples of how digital health has been transforming the operational workflow of biopharma and clinical research organizations [7]. Digital biomarker development has also been a key application of digital health in the biopharma and clinical research spaces for developing new end points that have not previously been possible to assess or existing end points that can be measured in new and possibly better ways [8]. Recently, Green et al have reported a digital biomarker for detecting patients with obstructive hypertrophic cardiomyopathy using signals collected from a wrist-worn wearable device and machine learning algorithms [9]. Other example applications in therapeutic areas including atrial fibrillation and obstructive sleep apnea [10-12] can potentially be used for evaluating the safety and efficacy of the drugs and interventions; for screening and referring patients who can benefit from lifesaving therapies; and as a companion tool for dose titration, decision support, and disease management. In summary, wearable and connected device technologies are evolving rapidly and may soon become the new standard in health monitoring to improve and accelerate the way drugs are developed, new therapies are identified, and patients are cared for.

**Figure 1:**
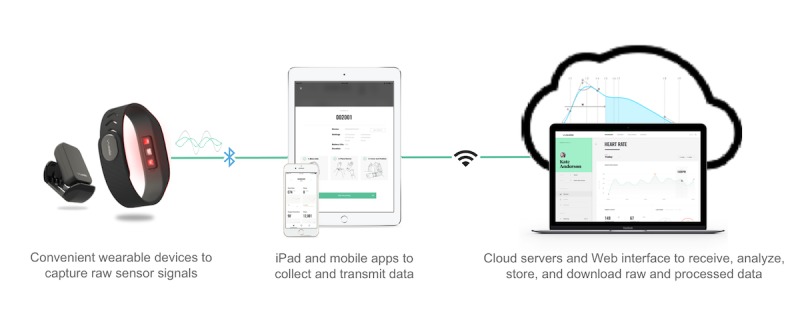
The data collection platform comprises wearable and connected devices that use low energy Bluetooth technology to communicate with the mobile and computer tablet software apps to collect and transmit raw sensor data and cloud servers; algorithms; and a Web interface to analyze, store, and access data.

The new era of personalized digital medicine requires devices that are convenient and engaging to the patient and have validated accuracy to enable a streamlined collection of biosignals and clinically meaningful health metrics. Many of the currently available remote monitoring options, including electrocardiography (ECG)-based Holter devices, event recorders, or mobile cardiac telemetry devices, require frequent replacements to extend their utility beyond 7 to 14 days and often cause discomfort to the patient or complications because of their intrusive nature. There is consensus on neither the benefit of continuous monitoring with implantable loop recorders on patient outcomes nor on whether these invasive devices are suitable for large-scale population-level disease surveillance and health screening. Advances in semiconductor and sensory technologies, in particular, the wide adoption of near-infrared (IR) light spectroscopy or photoplethysmography (PPG) in consumer health and clinical applications, along with mobile and cloud computing technology have generated a new class of wearable devices for health data collection and monitoring. Leveraging sophisticated signal processing algorithms such noninvasive devices allow near-real-time, continuous, and long-term monitoring of several key physiological parameters including heart rate (HR), heart rate variability (HRV), and respiration rate (RR) [13]. This study focuses on the performance evaluation of such a wrist-worn PPG device (Wavelet wristband, Wavelet Health, Mountain View, USA) and its data collection platform, which is shown in Figure 1.

### Measuring Health: Vital Signs

Measurement of vital signs enables detection and monitoring of a number of conditions and diseases. Continuous HR monitoring is critical to the management of cardiovascular disease as elevated HR is an independent predictor of cardiovascular events, mortality, and hospitalization for worsening heart failure [14,15]. HRV is another clinically important metric often regarded as a measure of neurocardiac function and homeostasis [16]. Fluctuations in beat-to-beat timing arise from the interaction of different physiological systems including the heart, brain, and autonomic nervous system in healthy and diseased states. Although the study of HRV has been a topic of active research for over two decades [17,18], there are still inconsistencies in the literature probably because of methodological differences between studies [19-21]. Several investigators found that relatively high resting HRV is indicative of a healthy, resilient, and responsive nervous system regulating the heart’s activity, whereas reduced HRV is associated with unbalanced, sympathetic, and parasympathetic activity negatively affecting cognitive performance [22], physical training capacity [23], congestive heart failure [24,25], multiple sclerosis [26], Guillain-Barre syndrome [27], and diabetic neuropathy [28]. In contrast, other studies suggest a lack of a clear correlation between HRV and other biological and lifestyle factors [19,29,30].

RR, an often overlooked vital sign, enables early detection of life-threatening diseases, such as sleep apnea [31], pneumonia [32], sudden infant death syndrome [33,34], or chronic obstructive pulmonary disease [35]. As abnormal RR is predictive of a future critical illness [36,37], continuous monitoring would provide clinicians with a real-time indicator of their patients’ health. Current methods for measuring RR include manually counting chest movements, estimated RR from ECG patch devices, a spirometer, and capnography monitors [38]. These methods do not allow long-term continuous monitoring because of the need for manual supervision, cost, or discomfort to the patient. Alternatively, RR can be estimated from PPG devices by leveraging 3 signal processing methods: baseline wander, amplitude modulation, and frequency modulation—each stem from 3 main physiological mechanisms: changes in tissue blood volume, stroke volume, and respiratory sinus arrhythmia, respectively, caused by intrathoracic pressure changes during respiration [39-41]. Several PPG-based RR algorithms have been reported in the literature with variable levels of accuracy, that is, mean error of approximately 1 to 6 breaths per minute (brpm), depending on which combination of signal processing methods is incorporated in the algorithm formulation [42,43]. Birrenkott et al demonstrated the importance of establishing proper signal qualification methods to achieve accurate RR estimations from PPG signal [44]. In this study, we present the validation of a PPG-based RR estimation algorithm that combines the frequency modulation and baseline wander methods along with threshold-based respiratory signal qualification.

It is worthwhile to mention that unlike ECG devices, PPG technology does not measure the electrical activity of the heart but measures the changes in pulse volume. Therefore, the interbeat intervals of the PPG can deviate from RR intervals of the ECG, particularly during physical activity and some mental stressors [45] and in patients with peripheral arterial disease and structural heart problems [29]. However, as the standard of care for monitoring of vital signs requires continuous long-term measurements of HR and HRV rather than pulse rate and pulse rate variability, this study evaluates the performance of the Wavelet wristband to estimate HR and HRV in healthy subjects at the resting state.

### Objective

The aim of the study was to evaluate the accuracy of resting HR, HRV, and RR estimates of the Wavelet wristband and to benchmark its performance as a wrist-worn PPG device compared with gold standard reference devices. In addition, the signal quality of the Wavelet wristband is compared with a reference device.

## Methods

### Wristband Technology

The core technology of the Wavelet wristband relies on PPG, that is, an optical technique for detecting blood volume changes within the microvascular bed to estimate physiological parameters [46]. PPG has been used since the early 1960s, particularly for pulse oximetry, which is the standard-of-care tool for measuring peripheral arterial oxygen saturation (SpO_2_) [47]. By positioning a light sensor and a light-emitting diode (LED) on the same plane, that is, the reflectance-type sensor configuration, wrist-worn PPG devices can perform measurements from the skin surface [48]. Light emitted by the LEDs into the wrist is mostly absorbed by the underlying tissue. The reflected light is captured by a photodiode, which is sampled many times within a second to construct the PPG signal. The absorption of light varies with the changes in pulsatile arterial blood flow and generates a time-varying pulse waveform [47]. This signal can be recorded, transmitted, and used as a noninvasive longitudinal measurement of the underlying blood volume changes. Due to its good tissue penetration characteristics [49], PPG devices often rely on IR light for estimating the relative volumetric changes in the microvascular bed because of pulsatile blood flow. Using the IR signal, several biometrics including arterial pulsatility, HR, HRV, RR, and vascular tone along with others related to the cardiovascular and autonomic nervous systems can be computed noninvasively [13]. When paired with the IR signal, the red light signal enables estimating SpO_2_, which is known as pulse oximetry.

**Figure 2:**
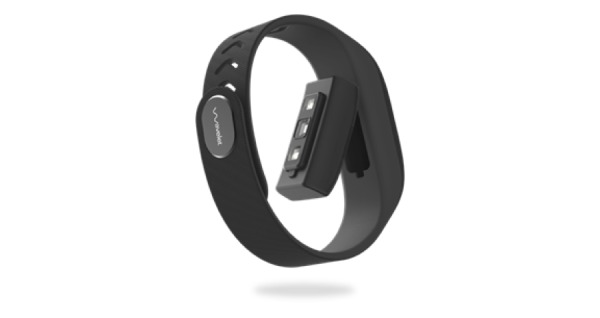
The Wavelet wristband is a configurable photoplethysmography and motion sensing device placed on the wrist to collect pulse wave signal, estimate vital signs, and physical activity. The sensor carriage can be removed from the plastic band.

During the measurement, the wristband is placed snugly on the arm above the wrist bone. The wristband comes with a removable sensor carriage and a plastic band as shown in Figure 2. The sensor carriage contains LEDs of 2 wavelengths and an optical sensor along with a battery and an inductive charging coil. The LEDs fire at a rate configurable between 20 and 95 Hz driven by a submillisecond resolution low-jitter external clock signal. A fully integrated analog front end receives and digitizes PPG signals. In addition, the wristband is capable of collecting inertial motion data using the 3-axis accelerometer and 3-axis gyroscope built into the sensor carriage. The sampling rate and duty cycle of the light and motion sensors are configurable. For this validation study, light sensor data were collected at 86 Hz and the motion data were collected at 10 Hz. Raw PPG and motion signal data collected from the wrist were transferred by the mobile app to the cloud server, where it is processed by signal processing and machine learning algorithms.

The general workflow of the algorithms developed for the extraction of HR, HRV, and RR from the PPG signals collected from the wrist is summarized below:

1. Segments of the PPG signal containing artifacts related to wrist movement are removed.
2. The PPG signal is segmented into heartbeats:
  - Peaks are detected in the PPG signal using a wavelet transform–based peak detection algorithm [50].
  - Peaks representing systolic onsets of beats are selected from this list of peaks by time and frequency domain heuristics. The location of the peaks is refined using interpolation techniques to improve the signal time resolution.
  - Beats are extracted from the PPG signal by taking segments from one systolic onset to the next and performing additional qualification.
3. From each heartbeat, HR and a number of other morphological features are extracted [51,52].
4. RR is measured by a combination of analyses of baseline wander and frequency modulation [41]:
  - In the time domain, inspiration and expiration are detected by a wavelet transform–based peak detection algorithm [52].
  - In the frequency domain, HR modulation because of respiration is measured by tracing the respiration ridge in the continuous wavelet transform of the HR signal [53].
5. HRV is estimated using the standard deviation (SD) of the normal-to-normal (SDNN) interbeat intervals and also the root mean square of the successive differences (RMSSD) between adjacent interbeat intervals over the course of the signal [54].

The beat segmentation approach enables the output of processed metrics on a beat-to-beat level, which is exemplified in the results section.

### Study Design

Healthy subjects (n=35) with no known cardiovascular conditions were recruited for the validation study. Participants were asked to determine their skin type using the Fitzpatrick questionnaire [55] and provided their height, weight, age, and gender. Before each test, subjects rested in a seated position for 15 min to ensure the measurement of the resting HR [56]. Demographics of the participants are summarized in Table 1. The study was approved by the institutional review board of San Jose State University. Written informed consent was obtained from all subjects.

The parameters estimated by the wristband were compared with the simultaneously recorded gold standard measurements from the ECG and spirometry sensors. For these measurements, a BIOPAC MP36 system (BIOPAC, Goleta, CA, USA) was set up to acquire ECG and spirometry data. ECG (LEAD110A and ECG100C, BIOPAC, Goleta, CA, USA) was acquired at a rate of 2000 Hz while the subject was at rest in a seated position. Spirometry data were acquired simultaneously and at the same rate using a handheld airflow transducer (SS11LA, BIOPAC, Goleta, CA, USA) connected to the BIOPAC system. Subjects were instructed to breathe through a mouthpiece while wearing a nose clip. Before the measurements, the ECG sensor was calibrated as per the manufacturer’s instructions [57]. No digital filtering was applied to the raw ECG and airflow data. Raw data were exported and analyzed to obtain HR and other metrics. A Nonin finger-clip pulse oximeter (8000 AA, Nonin, Plymouth, USA) was placed on the subject’s right index finger to compare the quality of the PPG signal recordings of the Wavelet wristband with a typical clinical grade finger-clip PPG device. For each test, 2 wristbands were placed on each participant, 1 on each wrist. Each test lasted between 2 and 4 min. For several subjects (n=12), the test was halted before the 3-min mark because of discomfort while breathing into the spirometer. Each subject repeated the test twice with 5 min rest in between. Overall, 70 tests were conducted each with 2 wristbands and reference ECG, spirometer, and finger-clip pulse oximeter device recordings. The synchronous recordings from ECG, Nonin, and Wavelet wristband devices were aligned manually based on time stamps and agreement of interbeat intervals, although a small misalignment was inevitable because of the lacking information on the pulse transit time.

### Signal Quality Analysis and Statistical Methods

Once each test session is recorded by the wristband and transferred to the cloud server, the algorithms described above calculate the beat-to-beat metrics. For the analysis, we separated each signal into 60-second nonoverlapping measurements and calculated HR, HRV, and RR for each of these windows to compare with HR, HRV, and RR of corresponding windows of the ECG signal. As PPG signals are inherently sensitive to motion and light artifacts, the biometrics algorithm evaluates each beat’s signal quality based on multiple heuristics including short-time Fourier transform, motion, and correlation with other beats. If not enough good-quality beats are found in the signal, the algorithm may not output some of the biometrics for a particular 60-second window. In this study, each qualified biometric assessment over a 60-second window was referred to as a valid measurement. Left and right wrists’ recordings were analyzed independently. The total number of valid sessions and the valid measurements qualified by the algorithm for each biometric are provided in the Results section.

To evaluate the signal quality of the Wavelet wristband, the harmonic-to-noise ratio (HNR) computed from the IR signal of the wristband was compared with that of the Nonin finger-clip pulse oximeter. HNR is calculated using the autocorrelation method described by Boersma et al [58]. This frequency-based signal quality assessment method yields an objective measure of the periodicity of the PPG signal from the maximum frequency of the signal’s normalized autocorrelation function [58]. The HNR is computed for 6-second overlapping windows centered 1 second apart. The average of all HNR windows is reported as the HNR of the 60-second recording. Recordings that fail to meet a threshold HNR level do not qualify for vital sign analysis. It is important to note that the HNR criterion assumes that all signals other than the signal of interest are noise. Therefore, to estimate HNR of the PPG signal accurately, a preprocessing step removes other physiological contributions to the signal such as respiration. The HNR reported herein follows the cardiovascular component of PPG signal after using a bandpass filter with lower and upper limits set to 0.3 Hz and 10 Hz, respectively.

To compare the HNR of the Wavelet wristband with the finger-clip pulse oximeter, we present the mean and SD of HNR for both devices for each valid measurement as well as box plots to compare distributions. The reference HR and HRV were computed from the reference ECG measurements using the Python BioSPPy biosignal processing toolbox [59], and Python is used for all statistical analysis. Ectopic and other non-normal beats were detected by a median absolute deviation–based outlier detection method and removed without replacement. An interbeat interval is considered an outlier if its logarithm is more than 6.25 median absolute deviations away from the median logarithm of interbeat intervals in the full recording. The use of logarithm and the threshold are determined from the visual inspection of the interbeat interval distribution. To compare the biometric estimates by the wristband with those measured in the same time window by the ECG and spirometer, Pearson correlation coefficients along with Bland-Altman plots and Bland-Altman limits of agreement are presented [60]. The Bland-Altman limits of agreement took into account multiple observations collected over time from the same set of individuals using 2 reference devices [61]. The effect of the averaging window size on the accuracy of HR estimates and the variation in biometric measurements collected from the left versus right wrists were also evaluated.

## Results

### Participant Demographics

Study participant demographics are summarized in Table 1. The average and SD of height, weight, and age were 172 cm (SD 10), 74 kg (SD 18), and 25 years (SD 4), respectively. The Fitzpatrick score indicates a good coverage of light, medium, and dark skin tones, with a slight skew to the darker pigmentation range.

### Photoplethysmography Signal Qualification

**Figure 3:**
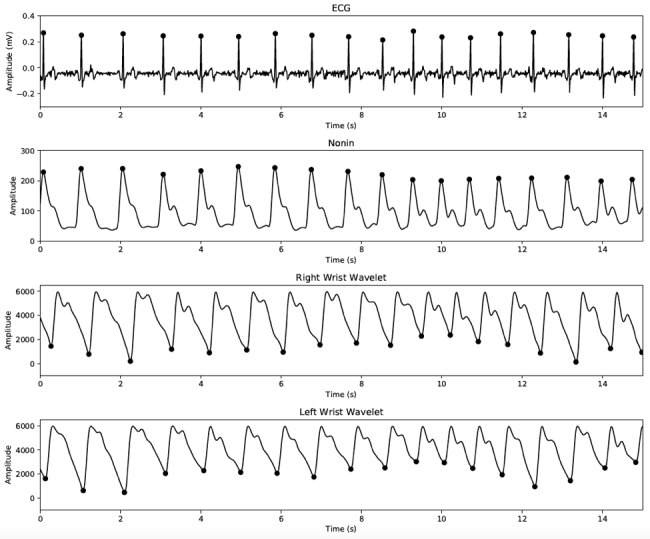
Signal traces recorded simultaneously from electrocardiography (ECG), Nonin finger-clip pulse oximetry device, and 2 Wavelet wristbands placed on the left and right wrists. Peaks (ECG and Nonin) or valleys (Wavelet) are marked. Signs were aligned based on time stamps and agreement of interbeat intervals in the absence of accurate pulse transit time information.

**Figure 4:**
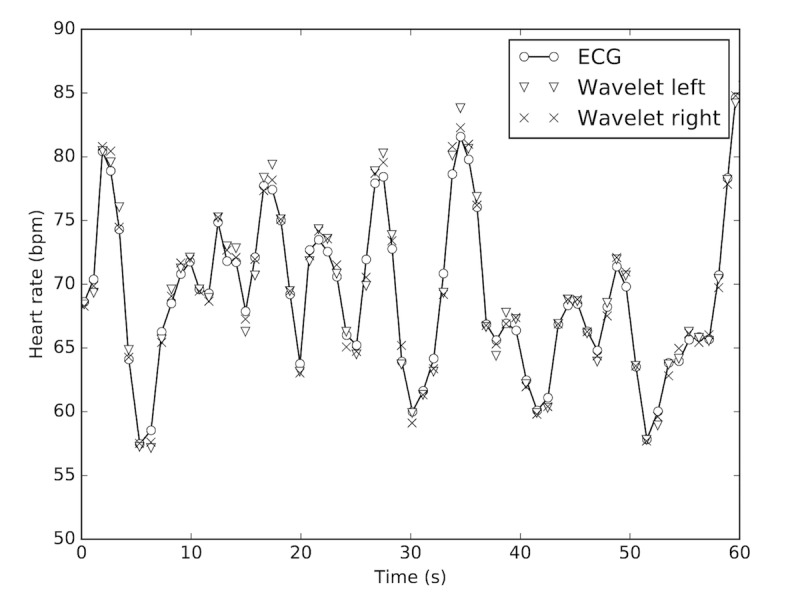
Representative instantaneous heart rate (HR) estimates from the wristbands on the left and right wrists show good agreement with the electrocardiography (ECG) HR measurements. Note that the beat-level HR data are computed in the background to estimate 60-second window HR averages reported in this study. Wavelet 1 and 2 labels refer to the wristband placed on the left and right wrists, respectively.

The PPG signals obtained from the Wavelet wristband have a physiological morphology similar to those collected from the Nonin finger-clip device, as shown in Figure 3. Compared with the reference finger-clip PPG measurements, the diastolic peak is located closer to the systolic peak and the diastolic decay is steeper, in agreement with the earlier studies comparing different measurement sites of PPG [62] and arterial pressure [63]. Figure 4 shows the continuous HR estimate of the Wavelet wristband, indicating strong agreement across right and left wrists and with the reference ECG device.

**Table 1:** Participant demographics (N=35).

Characteristics		Value
Height (cm), mean (SD)		172 (10)
Weight (kg), mean (SD)		74 (18)
Age (years), mean (SD)		25 (4)
Gender (female), n (%)		16 (46)
**Fitzpatrick score^a^, n (%)**		
	Type I	2 (6)
	Type II	6 (17)
	Type III	12 (34)
	Type IV	14 (40)
	Type V	1 (3)

^a^Self-reported Fitzpatrick scores classify subjects’ skin tones as follows: type I, pale white; type II, white; type III, cream white; type IV, moderate brown; and type V, dark brown.

**Figure 5:**
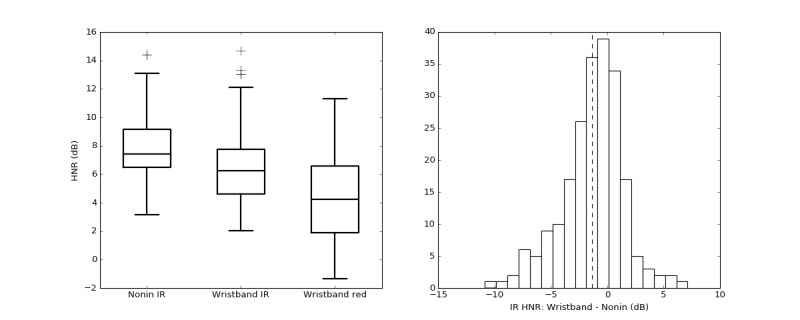
Boxplot comparison (left) of average harmonic-to-noise ratio (HNR) estimated over 60-second nonoverlapping windows for each photoplethysmography wavelength: Nonin finger-clip pulse oximeter (Nonin IR), the wristband infrared (Wavelet IR), and the wristband red (Wavelet red). The histogram for pairwise difference of average HNR between Wavelet IR and Nonin IR signals (right). IR: infrared.

To assess the quality of the signal at each LED wavelength, average HNR values for each 60-second nonoverlapping window were computed for signals collected by the Nonin finger-clip PPG device and the wristband. Table 2 shows the mean (SD) of HNR as 7.7 (SD 2.0), 6.4 (SD 2.2), and 4.7 (SD 2.7) for Nonin IR, Wavelet IR, and Wavelet red, respectively. The distribution of HNR for each group is illustrated in Figure 5 using boxplots. Note that not all Wavelet wristband recordings had a corresponding Nonin recording available because of equipment availability on the test day. The difference histogram in Figure 5 illustrates the pairwise mean difference (with 1 SD) between the wristband IR HNR and Nonin HNR as −1.34 (SD 2.75) dB. Thus, the wristband IR HNR is slightly lower than that of the finger-clip sensor.

In this study, a total of 366 min of PPG signal recordings were collected from 35 subjects. Approximately 12.0 % (44/366) of the PPG recordings were flagged invalid because of subtle arm motions during the test. In addition, 74 measurements from 23 test sessions failed to meet the desired HNR level and did not qualify to vital sign analysis. The signal processing algorithm generated valid HR, HRV SDNN, HRV RMSSD, and RR measurements for 78.9% (254/322), 76.7% (247/322), 77.3% (249/322), and 39.8% (128/322) of the signals, respectively. Importantly, the signal quality preprocessing step disqualified all readings from 2 type II and 1 type I subjects. Furthermore, it was later found that for 1 subject, the ECG probe was dislocated; therefore, no valid ECG reference data were collected. The number of valid vital sign measurements for which the corresponding reference data exist is shown in Table 3.

### Heart Rate Validation

**Figure 6:**
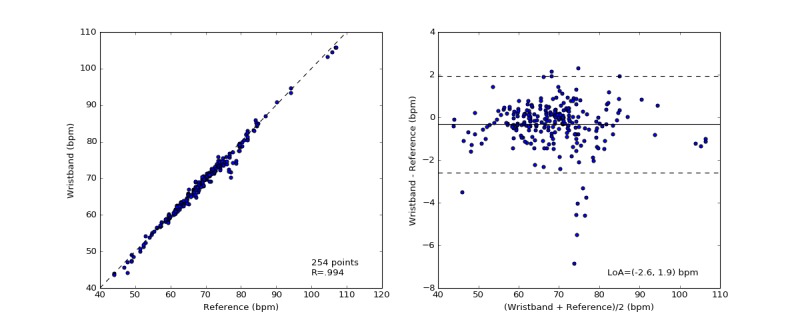
The distribution of measured heart rate (HR) by the electrocardiography (ECG) and the wristband (left). The Pearson correlation coefficient is shown in the lower-right corner of this scatter plot. Bland-Altman plots of the absolute error of HR between the wristband and the simultaneously recorded ECG measurements versus the mean of the measurements in beats per minute (right). The solid black line indicates the mean difference. The dotted lines mark the 95% limits of agreement (LoA) at −2.6 and 1.9 bpm.

Table 3 shows the mean absolute error and mean absolute percentage error of the HR, HRV, and RR estimates of the Wavelet wristband compared with the reference devices. Across 254 measurements of 60-second nonoverlapping windows, the mean pairwise absolute error of HR was 0.7 beats per minutes or bpm (SD 0.9; 0.9%, SD 1.3) against the reference ECG. Figure 6 shows the distribution of ECG and Wavelet HR estimates with scatter and Bland-Altman plots. The Pearson correlation coefficient (R) of HR between wristband estimates and reference ECG measurements was .994, and the mean difference (bias) between Wavelet and ECG HR (with 95% CI) was −0.32 bpm (SD 0.13). All measurements stay within 5% absolute percent error, except one outlier for which the Wavelet wristband underestimated the HR by 7% (6.8 bpm). The Bland-Altman ratio, that is, the ratio of 1.96 x SD divided by the mean of the pairwise measurement means, is equal to 0.03, which indicates good agreement between measurements [60].

**Table 2:** The mean and SD of the average harmonic-to-noise ratio for the photoplethysmography recordings collected from the Wavelet wristband and reference Nonin finger-clip pulse oximeter.

Photoplethysmography signals	Valid sample size^a^		Mean HNR^b^ (SD)
	Measurements, n	Subjects, n	
Nonin IR^c^ (dB)	216	28	7.7 (2.0)
Wavelet IR (dB)	266	33	6.4 (2.2)
Wavelet red (dB)	266	33	4.7 (2.7)

^a^Valid sample size: the number of measurements and subjects that were eligible for signal quality analysis.

^b^HNR: harmonic-to-noise ratio.

^c^IR: infrared.

**Table 3:** The accuracy of the biometric estimates (mean, SD) of the Wavelet wristband compared with the reference electrocardiography and respirometer measurements.

Measurement	Valid sample size^a^		Mean absolute error (SD)	Mean absolute percentage error (SD)	Mean error (SD)	Pearson correlation
	Measurements, n	Subjects, n				
HR^b^ (bpm^c^)	254	31	0.7 (0.9)	0.9 (1.3)	−0.3 (1.1)	.994
HRV SDNN^d^ (ms)	247	31	7 (10)	11 (13)	−1 (12)	.907
HRV RMSSD^e^ (ms)	249	31	11 (12)	28 (30)	3 (16)	.924
RR^f^ (brpm^g^)	128	26	1 (1)	2.5 (2.5)	1 (2)	.863

^a^Valid sample size refers to the number of measurements and subjects where both valid wristband and reference data were available.

^b^HR: heart rate.

^c^bpm: beats per minute.

^d^HRV SDNN: heart rate variability standard deviation of normal-to-normal intervals.

^e^HRV RMSSD: heart rate variability root mean square of successive differences.

^f^RR: respiratory rate.

^g^brpm: breaths per minute.

**Figure 7:**
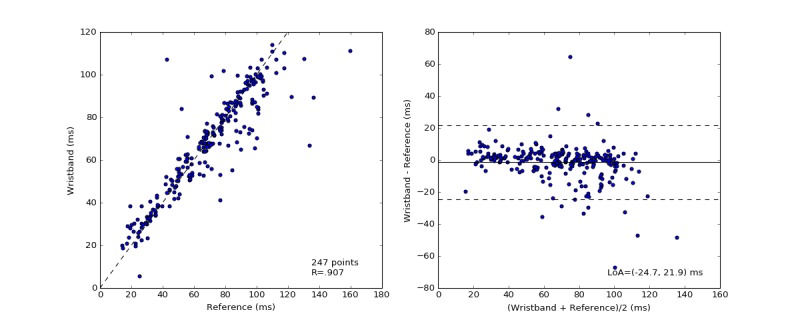
The distribution of measured heart rate variability standard deviation of normal-to-normal intervals (HRV SDNN) by the electrocardiography (ECG) and the wristband (left). The Pearson correlation coefficient is shown in the lower-right corner of this scatter plot. Bland-Altman plots of the absolute error of HRV between the wristband and the simultaneously recorded ECG measurements versus the mean of the measurements in milliseconds (right). The solid black line indicates the mean difference. The dotted lines mark the 95% limits of agreement (LoA) at −25 and 22 ms.

### Heart Rate Variability

**Figure 8:**
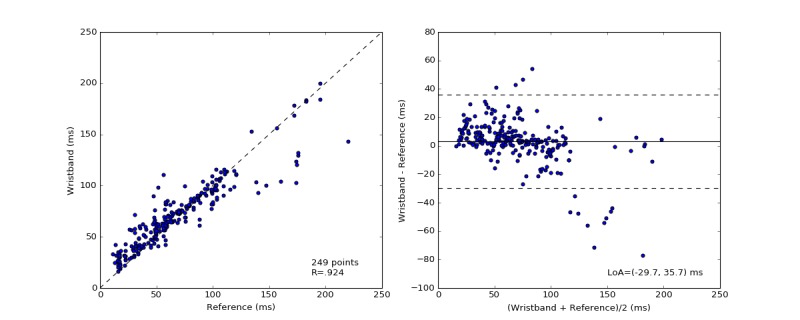
The distribution of measured heart rate variability root mean square of successive differences (HRV RMSSD) by the electrocardiography (ECG) and the wristband (left). The Pearson correlation coefficient is shown in the lower-right corner of this scatter plot. Bland-Altman plots of the absolute error of HRV between the wristband and the simultaneously recorded ECG measurements versus the mean of the measurements in milliseconds (right). The solid black line indicates the mean difference. The dotted lines mark the 95% limit of agreement (LoA) at −30 and 36 ms.

The SDNN and RMSSD HRV are computed over 60-second nonoverlapping windows for ECG and Wavelet wristband recordings. The mean absolute errors for SDNN and RMSSD were estimated as 7 ms (SD 10; 11%, SD 13) and 11 ms (SD 12; 28%, SD 30), respectively, as shown in Table 3. The mean difference (with 1 SD) between Wavelet and ECG-based SDNN and RMSSD was −1 ms (SD 12) and 3 ms (SD 16), respectively. The relationship between the Wavelet SDNN HRV estimates to the ECG is visualized with scatter plots as well as with Bland-Altman plots (Figures 7 and 8). Pearson correlation coefficients for HRV SDNN and RMSSD were estimated as .907 and .924, respectively. The Bland-Altman ratios for HRV SDNN and RMSSD were 0.35 and 0.42, which indicate strong correlation between the wristband HRV estimates and the reference measurements. Relatively lower correlation for RMSSD estimates is attributed to the outliers at the high RMSSD range (>150 ms).

### Respiration Rate

**Figure 9:**
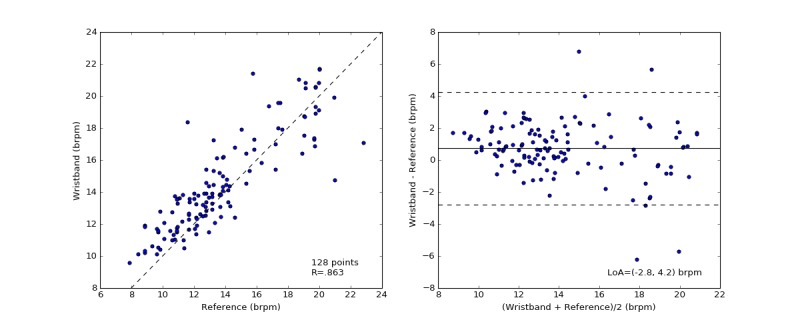
The distribution of measured respiration rate (RR) by spirometer and the wristband (left). Each data point represents the average RR over a 60-second nonoverlapping measurement window. Bland-Altman plots of the absolute error of RR between the wristband and the simultaneously recorded control measurements versus the mean of the measurements in breaths per minute (right). The solid black line indicates the mean difference. The dotted lines mark the 95% limit of agreement (LoA) at −3 and 4 brpm.

Figure 9 shows the comparison of the RR estimates of the wristband with the reference spirometry measurements. The Pearson correlation coefficient was .863, and the mean difference (bias with 1 SD) between Wavelet and spirometer RR was 1 brpm (SD 2). The pairwise mean absolute error is 1 brpm (SD 1; 2.5%, SD 2.5) as shown in Table 3.

To assess the agreement between the HR and HRV computed by a Wavelet wristband on the left and right wrists, the mean absolute error is computed for subjects where both valid simultaneous left and right wrists’ readings are available (n=23). Table 4 shows that the mean absolute difference between the right and left wrists’ measurements was 0.6 bpm (SD 0.8), 6 ms (SD 10), 9 ms (SD 10), and 1 brpm (SD 2) for HR, HRV SDNN, HRV RMSSD, and RR, respectively.

### Influence of Recording Window Duration on Biometric Estimation Accuracy

For certain use cases, the estimation of biometrics over durations shorter than 60 seconds may be desirable. To assess the accuracy of the biometric estimations over shorter recording durations, the absolute error and absolute percentage errors from the reference were computed by reanalyzing the test and reference signals over 45 and 30 seconds long windows. Results shown in Table 5 indicate that the mean absolute error in HR remains stable, that is, within 1 bpm, as the recording duration shortens from 60 to 30 seconds. Similarly, mean absolute error in HRV RMSSD estimates remained at 12 ms without displaying dependence on recording duration. However, the mean absolute error of SDNN was influenced by the recording duration. For 30-second recording duration, mean absolute error for RR estimations increased to 2 brpm (SD 2).

**Table 4:** The pairwise mean absolute difference between the left and right wristbands.

Measure	Valid sample size^a^ (pairs)		Mean absolute difference (SD)
	Measurements, n	Subjects, n	
HR^b^ (bpm^c^)	108	23	0.6 (0.8)
HRV SDNN^d^ (ms)	102	23	6 (10)
HRV RMSSD^e^ (ms)	101	23	9 (10)
RR^f^ (brpm^g^)	48	23	1 (2)

^a^Valid sample size refers to the number of measurements and subjects where two wristband recordings (pairs) from the same subject were available.

^b^HR: heart rate.

^c^bpm: beats per minute.

^d^HRV SDNN: heart rate variability standard deviation of normal-to-normal intervals.

^e^HRV RMSSD: heart rate variability root mean square of successive differences.

^f^RR: respiratory rate.

^g^brpm: breaths per minute.

**Table 5:** Mean absolute error and SD of biometrics estimations from reference measurements at different recording window durations.

Recording window duration	HR^a^ (bpm^b^)		HRV SDNN^c^ (ms)		HRV RMSSD^d^ (ms)		RR^e^ (brpm^f^)	
	Mean (SD)	n	Mean (SD)	n	Mean (SD)	n	Mean (SD)	n
60 (s)	0.7 (0.9)	254	7 (10)	247	11 (12)	249	1 (1)	128
45 (s)	0.8 (1.0)	381	7 (11)	361	11 (13)	364	1 (1)	163
30 (s)	1.0 (1.4)	559	8 (12)	516	11 (14)	520	2 (2)	218

^a^HR: heart rate.

^b^bpm: beats per minute.

^c^HRV SDNN: heart rate variability standard deviation of normal-to-normal intervals.

^d^HRV RMSSD: heart rate variability root mean square of successive differences.

^e^RR: respiratory rate.

^f^brpm: breaths per minute.

## Discussion

### Principal Findings

To access the clinical utility of wearable health devices, it is imperative to validate the accuracy of the metrics derived by these devices against gold standard measures and, importantly, to characterize their limitations. In this study, we evaluated the performance of the Wavelet wristband by comparing the accuracy of the estimated biometrics with the ground truth references. The mean absolute error of HR, HRV SDNN, HRV RMSSD, and RR was 0.7 bpm (SD 0.9), 7 ms (SD 10), 11 ms (SD 12), and 1 brpm (SD 1), respectively. Bland-Altman graphs demonstrate good agreement between the wristband biometric estimates with the reference measurements. These results indicate that the Wavelet wristband can estimate multiple vital signs in the resting state and provide a continuous noninvasive health monitoring solution as an alternative to other devices typically incorporating ECG.

To our knowledge, this study is the first of its kind exploring the PPG signal quality collected by a wrist-worn reflective PPG device as compared with more traditional devices placed on measurement sites such as the finger, where the subcutaneous tissue is perfused more strongly with dense microvasculature. Previously, several investigators reported accurate estimation of vital signs using PPG signal collected from the finger in comparison with ECG in healthy [64-68] and disease settings [69-71]. Investigations by Maeda et al [62] showed that relatively high signal strength can be obtained from the wrist but lacked signal-to-noise ratio assessments, which are essential for estimating accurate biometrics. In this study, we suggest HNR can be used to characterize PPG signal quality collected from different measurement locations, benchmark PPG devices, and aid in establishing signal quality standards in PPG research. Comparable HNR of the Wavelet wristband and Nonin finger-clip pulse oximeter devices indicates that good quality PPG signal can be collected from the wrist.

### Strengths and Limitations

Although both PPG and ECG signals convey physiological information, the underlying physiology of PPG stems primarily from hemodynamics rather than the electrical activity of the heart depicted in the ECG signal. The well-defined morphology of the ECG signal allows relatively simple extraction of beat-to-beat intervals in the absence of artifacts related to drift, electromagnetic, and biological interferences [66]. In contrast, PPG signal hosts inherently more rounded peaks and valleys and therefore requires more sophisticated algorithms to extract physiological measures. Similar to blood pressure, PPG signal morphology depends strongly on the timing of reflected waves from the downstream vasculature [72], which is negatively correlated with vascular stiffness and age [13]. It is reasonable to assume changes in pulse transmit time, which is due to within-subject vascular stiffness variations, add another layer of challenge to accurate extraction of salient features from the PPG signal and contribute, in part, to deviations reported herein from the reference device measurements.

PPG signal quality is affected by multiple factors including improper sensor-skin coupling because of device malposition, ambient light, pressure on skin, and biological factors (blood perfusion, tissue composition, and skin temperature) and is highly sensitive to motion [47,73]. To eliminate inaccurate readings from PPG devices, it is necessary to incorporate proper signal qualification checks and biometric-specific heuristics to the signal processing algorithm. Only then will the PPG device be able to estimate biometrics within the desired accuracy range while providing a sufficient number of biometric readings for the designed use case. In this study, 78% of HR measurements and only 40% RR measurements were qualified by the vital sign algorithms, as the latter is highly sensitive to artifacts. It is important to note that cloud computing and storage of raw signals enable retrospective processing of the physiological signals collected by digital health devices. Leveraging more advanced algorithms, this framework will allow further improvement of the accuracy and the number of the valid readings generated from the same signals.

In addition to postprocessing strategies, the choice of the PPG wavelength also impacts the quality of the PPG signal and accuracy of the computed metrics. This choice is a trade-off and depends on the targeted application but is usually between 510 and 940 nm, corresponding to green and IR lights, respectively. Measurements performed on light skins and at normal ambient temperature (around 20°C) have shown that reflected green light maintains a good signal-to-noise ratio during motion compared with IR [74], which is the main reason why many consumer devices that target ambulatory HR measurements use a green light source. The advantage of IR light over green light is that it is less sensitive to skin tone variations and perfusion level because of its better tissue penetration characteristics [49]. The darker the skin pigmentation, that is, higher melanin concentration, the harder it is to receive good signal with light wavelengths shorter than 650 nm. Furthermore, for individuals with relatively lower superficial skin perfusion, thicker skin, and larger wrist circumference or body mass indices, particularly in cold ambient conditions, blood microcirculation is significantly lower, and it becomes advantageous to reach deeper tissues. Therefore, to serve particularly for resting state biometrics monitoring across the population, the Wavelet wristband uses IR light as its primary light source.

As a limitation of this study, the biometrics reported herein were tested at the resting condition and lacked physical activity settings. It is well known that the accuracy of biometrics derived from PPG-based devices is affected by motion artifacts [75,76]. Recent studies evaluating HR estimates from several commercially available wrist-worn PPG devices reveal variable degrees of accuracy during physical activity [77,78]. Accuracy of the biometrics estimation during motion requires robust motion reduction algorithms [79-81], which is not incorporated in the current version of the product. Future research in this area is needed to preserve the original signal morphology and extract more information than just ambulatory HR. Another limitation is that the subjects recruited for the study have no known health conditions and come with restricted age range and skin tones available. The correlation between measurement error and Fitzpatrick score indicates higher deviations at the extremes. Further studies are needed to demonstrate the utility of the devices for larger more diverse populations. Moving forward, well-designed clinical studies are required to demonstrate the impact of new wearable and connected devices on clinical outcomes and to build real-world evidence for indications that benefit most from these new technologies.

### Conclusions

This study demonstrates that the Wavelet wrist-worn PPG device can estimate physiological measures including HR, HRV SDNN, HRV RMSSD, and RR within 0.6 bpm (SD 0.9), 7 ms (SD 10), 11 ms (SD 12), and 1 brpm (SD 1), respectively, of the reference ECG and spirometer devices. The quality of the PPG signal generated by the Wavelet wristband and the commercially available finger-clip pulse oximeter was evaluated at the identical conditions and quantified by HNR estimations as 6.40 dB (SD 2.16) and 7.70 dB (SD 1.99), respectively. Next-generation wearable and connected devices provide unprecedented means for continuous long-term remote health monitoring. Due to their noninvasive, convenient-to-patient, and engaging nature, these technologies will be gradually becoming part of our everyday lives and act as companion tools for clinical decision support, supplementing the established gold standard methods. This new streamlined health data collection modality will enable new and better ways to measure personal health, generate insights that are otherwise not available, and ultimately improve health care delivery.

## Abbreviations

bpm: beats per min
brpm: breaths per minute
ECG: electrocardiography
HR: heart rate
HRV: heart rate variability
HNR: harmonic-to-noise ratio
IR: infrared
LED: light-emitting diode
PPG: photoplethysmography
RR: respiration rate
RMSSD: root mean square of successive differences between adjacent interbeat intervals
SDNN: standard deviation of normal-to-normal intervals
SpO2: peripheral arterial oxygen saturation
